# *KENeV*: A web-application for the automated reconstruction and visualization of the enriched metabolic and signaling super-pathways deriving from genomic experiments

**DOI:** 10.1016/j.csbj.2015.03.009

**Published:** 2015-04-09

**Authors:** Eleftherios Pilalis, Theodoros Koutsandreas, Ioannis Valavanis, Emmanouil Athanasiadis, George Spyrou, Aristotelis Chatziioannou

**Affiliations:** a Metabolic Engineering and Bioinformatics Programme, Institute of Medicinal Chemistry and Biotechnology, National Hellenic Research Foundation, Athens, Greece; b Biomedical Research Foundation, Academy of Athens, Athens, Greece

**Keywords:** KEGG, Enrichment analysis, Molecular pathways, Gene expression, Microarrays, Next generation sequencing

## Abstract

Gene expression analysis, using high throughput genomic technologies,has become an indispensable step for the meaningful interpretation of the underlying molecular complexity, which shapes the phenotypic manifestation of the investigated biological mechanism. The modularity of the cellular response to different experimental conditions can be comprehended through the exploitation of molecular pathway databases, which offer a controlled, curated background for statistical enrichment analysis. Existing tools enable pathway analysis, visualization, or pathway merging but none integrates a fully automated workflow, combining all above-mentioned modules and destined to non-programmer users.

We introduce an online web application, named *KEGG Enriched Network Visualizer* (*KENeV*), which enables a fully automated workflow starting from a list of differentially expressed genes and deriving the enriched KEGG metabolic and signaling pathways, merged into two respective, non-redundant super-networks. The final networks can be downloaded as SBML files, for further analysis, or instantly visualized through an interactive visualization module.

In conclusion, *KENeV* (available online at http://www.grissom.gr/kenev) provides an integrative tool, suitable for users with no programming experience, for the functional interpretation, at both the metabolic and signaling level, of differentially expressed gene subsets deriving from genomic experiments.

## 1 Introduction

Gene expression analysis using DNA microarrays or next-generation sequencing (NGS) technologies is commonly employed, in order to derive lists of genes that are differentially expressed among various experimental conditions. Obtaining a comprehensive list of genes is routinely performed today, given the high-throughput biological experiments and the plethora of methods for further statistical analysis of the data obtained. The subsequent association of a list of genes with particular cellular functionalities, through the identification of specific molecular pathways, highlights the modular character of the cellular response to the change of conditions, for instance the chemical environment, disease states, or drug-induced effects [1]. From a Systems Biology perspective, it is becoming obvious how critical is to identify entire biological processes and complex interactions, found in various metabolic and signaling pathways, rather than only highlight isolated biological factors [2]. Moreover, the over- or under-expression of a single gene does not give significant information about the actual effect in cellular physiology, unless all known interactions are taken into account.

The most common approaches for pathway analysis are based on the statistical enrichment scores of various annotations [3], [4], [5], for instance Gene Ontology (GO) terms [6] and identifiers of pathway databases, for instance the *Kyoto Encyclopedia of Genes and Genomes* (KEGG) biological pathway database [7] and the *Molecular Signatures Database (*MSigDB) [3]. Such methods that exploit knowledge hosted in public repositories perform knowledge-based driven pathway analysis and are opposed to methods that use solely molecular measurements derived from an experiment [8]. Knowledge-based driven methods include over-representation analysis (ORA) methods, functional-scoring class (FCS) methods and pathway-topology (PT) based methods. ORA methods were developed firstly with GO terms emergence in order to cover the immediate need for functional analysis. Briefly, they use the statistical enrichment score in annotations found in various controlled vocabularies to statistically evaluate a fraction of genes in a particular pathway, which are found among the set of genes showing changes in expression. Examples of ORA tools are *Onto-express* [9] and *GoMiner* [10]. In FCS approaches, *GeneTrail* for example [11], a gene-level statistic is firstly computed using differential gene or protein expression, gene-level statistics for all genes in a pathway are then aggregated into a single pathway-level statistic and finally all statistical significance for all pathways are evaluated. PT-based methods [12] (e.g. *Pathway-Express* [13] and *ScorePAGE* [14]) incorporate additional pathway topology information included in the public databases in order to compute gene-level statistics, e.g. gene products that interact with each other in a given pathway, how they interact (activation, inhibition, etc.), and where they interact (cytoplasm, nucleus, etc.).

**Fig. 1:**
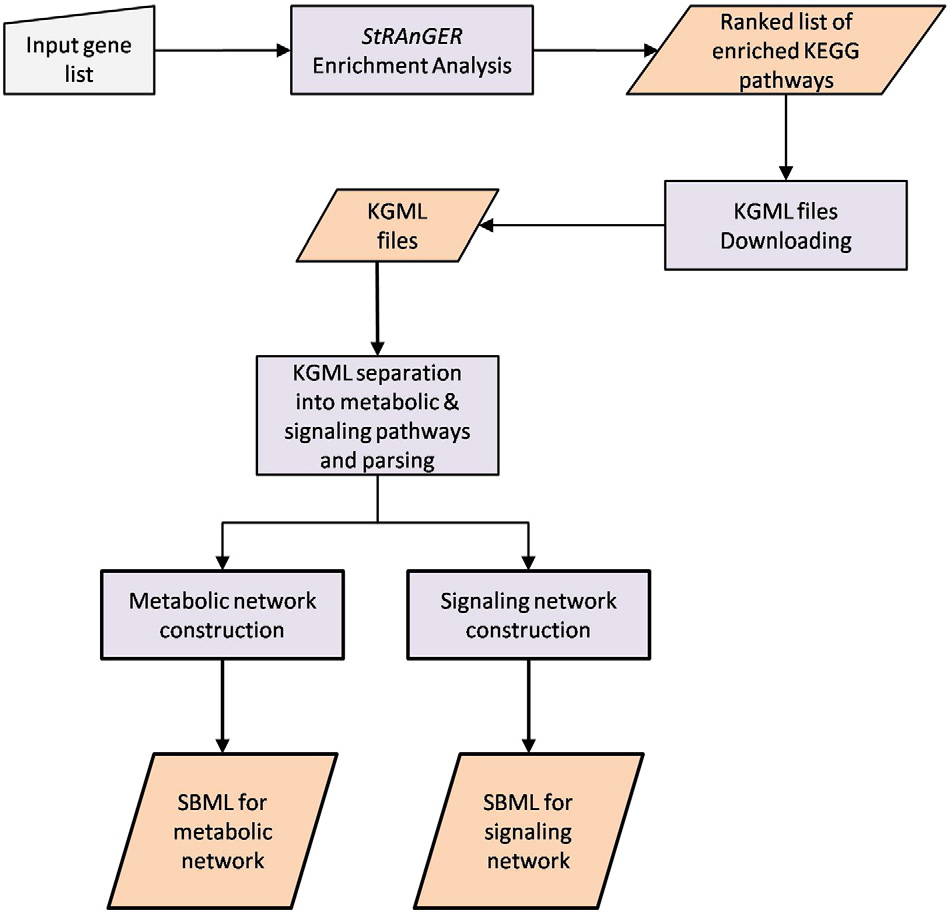
Overall application workflow.

In this work, we have developed an online web application based on the ORA approach, named *KEGG Enriched Network Visualizer* (*KENeV*), which exploits the widely used KEGG pathway database, in order to automatically derive and visualize the enriched molecular networks. KEGG is a library of molecular networks that has been widely used as a reference point for biological interpretation of large-scale datasets. The application takes as input a list of significant genes, from an expression analysis experiment, possibly accompanied by corresponding fold change measurements. The workflow [Fig. 1] starts with pathway enrichment analysis of the input gene list, using the *StRAnGER* algorithm [15], [16], which prioritizes statistically significant enrichments of terms that belong to a controlled annotation vocabulary, as for instance, Gene Ontology terms or the identifiers of KEGG pathways. Subsequently, the application identifies and separates the promoted pathways in two levels, metabolic and signaling (protein–protein, and protein–small molecule interaction). These pathways are automatically downloaded, without any intervention by the user, converted to SMBL format [19], merged into two super-pathways, a metabolic and a signaling one, and finally delivered in two respective SBML files that can be downloaded or visualized on-the-fly in an embedded visualization module. In addition to the two super-pathways, the application provides a gene-pathway mapping visualization that depicts the pathways in which each selected gene participates. Finally, the application is demonstrated using a list of genes that has been associated to Type I Diabetes Mellitus and was found in the Autworks database [17].

## 2 Materials and methods

### 2.1 Pathway enrichment analysis

The pathway enrichment analysis is performed by the *StRAnGER* algorithm, described in detail in [15], [16] which employs a combination of a parametric (Hypergeometric) and a non-parametric statistical test (bootstrap resampling) [18]. The algorithm initially uses the Hypergeometric test to assess the over-representation of KEGG pathways to the input gene list, and finally ranks the over-represented pathways. However, the final pathway ranking is based on a non-parametric, empiric algorithm which avoids assumptions about the distribution of term enrichments and thus can be adapted to any set of terms. With the bootstrap resampling, *StRAnGER* avoids the utilization of multiple test correction approaches (Bonferroni, FDR) that are very restrictive and problematic in regard to the finite nature of annotation vocabularies. The main problem of these methodologies is that they tend to promote enrichments yielding very low *p*-values (close to 0), but holding a very low biological content (e.g. enrichments such as 2/2 and 1/2). Bootstrapping is a non-parametric, empirical alternative to multiple test correction, which provides a corrected measure for the statistical significance of the enrichments based on their frequencies of observation. Instead of adjusting the *p*-values, the algorithm reorders the initial distribution and prioritizes the less frequently observed enrichments. The enrichments are derived as statistically significant if they satisfy both the hypergeometric and the bootstrap thresholds, but they are ranked according to their bootstrap *p*-value. Hence, the algorithm prioritizes pathways with less frequent enrichments which tend to represent broader pathways or functions and, thus, are of stronger biological content. The final output of significant KEGG IDs is spanned by the KEGG pathways that are mapped to the significant elements.

### 2.2 XML parsing and merging

The statistically important pathways derived from the enrichment analysis are automatically downloaded from the REST interface of the KEGG database as KGML format files, which is the XML format used by KEGG. Subsequently, the KGML files are parsed (using the *ElementTree* Python XML parser) and their fields are stored as respective objects. Different types of compounds (simple molecules, glycans, drugs, proteins, enzymes) and relations (metabolic reactions, signaling cascades etc.) are detected using the KEGG annotation fields and are stored in the database. Each compound is then mapped to a unique node and to all reactions in which it participates. Finally, two merged networks are constructed using the *libsbml* library [19], [20], one containing only the metabolic reactions and another containing all other interactions. In the case of the metabolic reactions, the corresponding genes are stored as reaction modifiers, whereas in the case of the signaling network, the type of interaction (e.g. phosphorylation, protein–protein interaction, gene expression relation) is stored as the corresponding reaction name. Finally, two distinct SBML files are constructed, which do not contain redundancies.

### 2.3 Implementation

The application was implemented in the *Web2Py* framework, in *Python* language. A *NoSQL* database (*MongoDB*), was used for the storage of KEGG data (organism–gene, organism–pathway and gene–pathway mappings). The application was programmed to automatically download the relevant files on a weekly basis, using the KEGG REST API (freely accessible for academic use). The Network Visualization Tool was implemented using a custom made *JavaScript* algorithm complemented by *jQuery* (http://jquery.com/) and “*sigma.js*” (http://sigmajs.org/) *JavaScript* libraries.

## 3 Results

### 3.1 Application

The online application is available under the url http://www.grissom.gr/kenev. The form accepts as input a list of differentially expressed genes, derived from a genomic experiment, optionally accompanied by their expression fold change values (natural scale) in tab-delimited format. The user is able to select the examined organism species from the complete list of KEGG organisms (currently including 3677 species). Additionally, the user is able to choose the cut-off values of the two aforementioned statistical tests. On form submission, the pathway enrichment analysis is performed, resulting to a final ranked list of KEGG pathways. The user is then able to click on a link that performs the pathway merging step and yields the signaling and metabolic super-pathways. Remark that it is possible that the pathway list does not include both metabolic and signaling pathways. In this case, only one type of SBML file is returned. The SBML files are available for downloading, in order to be processed or visualized by external software, for instance by the popular programs *Cytoscape* [21] and *CellDesigner* [22]. An embedded interactive visualization module described next is provided, as well.

### 3.2 Visualization module

**Fig. 2:**
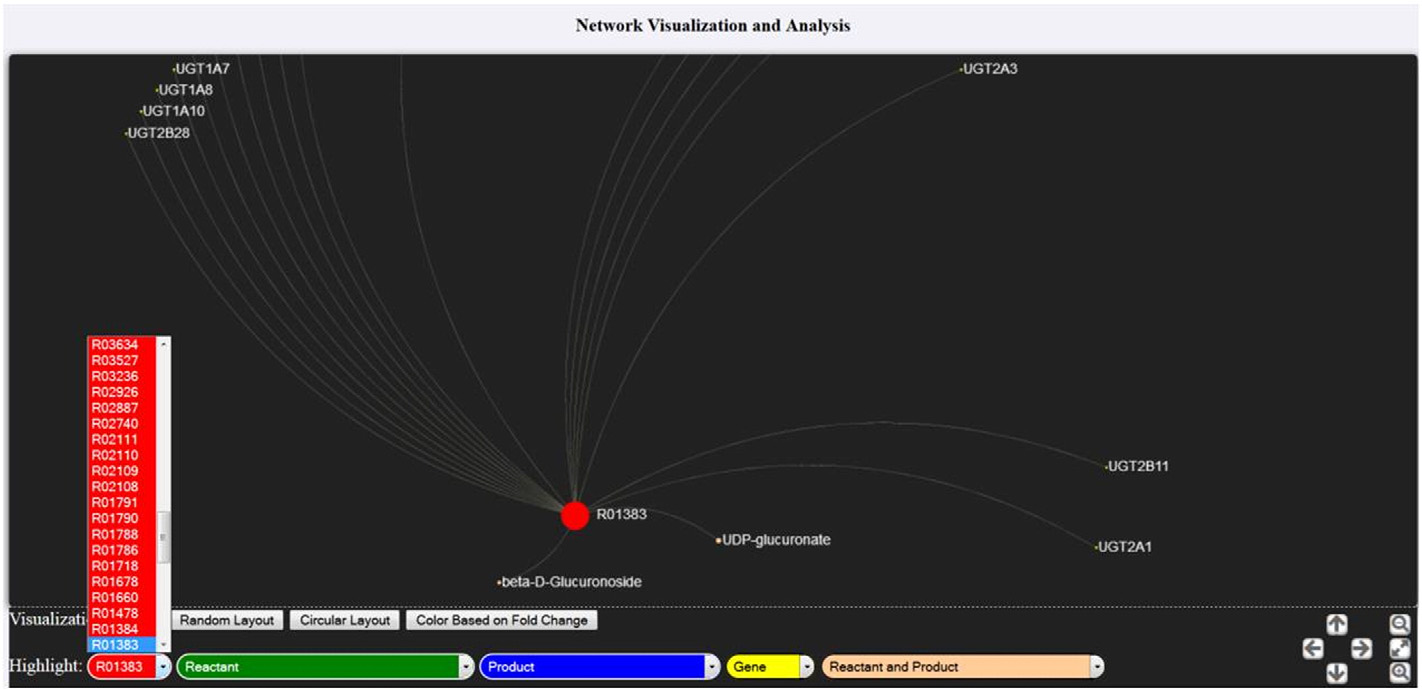
In this screenshot, visualization of a sample SBML output file is presented. Reaction with ID “R01383” has been randomly selected from the “Reaction” drop-down menu and the corresponding genes, reactants and products that comprise the selected reaction are highlighted.

For the case of the metabolic/signaling graphs using the generated SBML files (see Fig. 2), the network visualization can be performed using either a circular, a random or a force-directed (Fruchterman Reingold) layout. The node's size is proportional to the number of its first neighbors that interact with the specific node. Reactions, reactants, products, modifiers and both reactants and products nodes are colored and depicted in five corresponding concentric circles with red, green, blue, yellow and suntan, respectively. In addition, users are able to modify the node's colors based on the fold change of each gene if the respective values have been provided by the user. In the case of the latter color modification, green and red nodes correspond to up- and down-regulated genes, while nodes with no information are presented in gray color. Users are also able to locate and highlight a specific gene interactively, either by using the appropriate drop-down menu, or by clicking on the node. Finally, further visualization filters can be applied regarding node's labels and interactions, using the appropriate slider on the bottom of the web application.

**Fig. 3:**
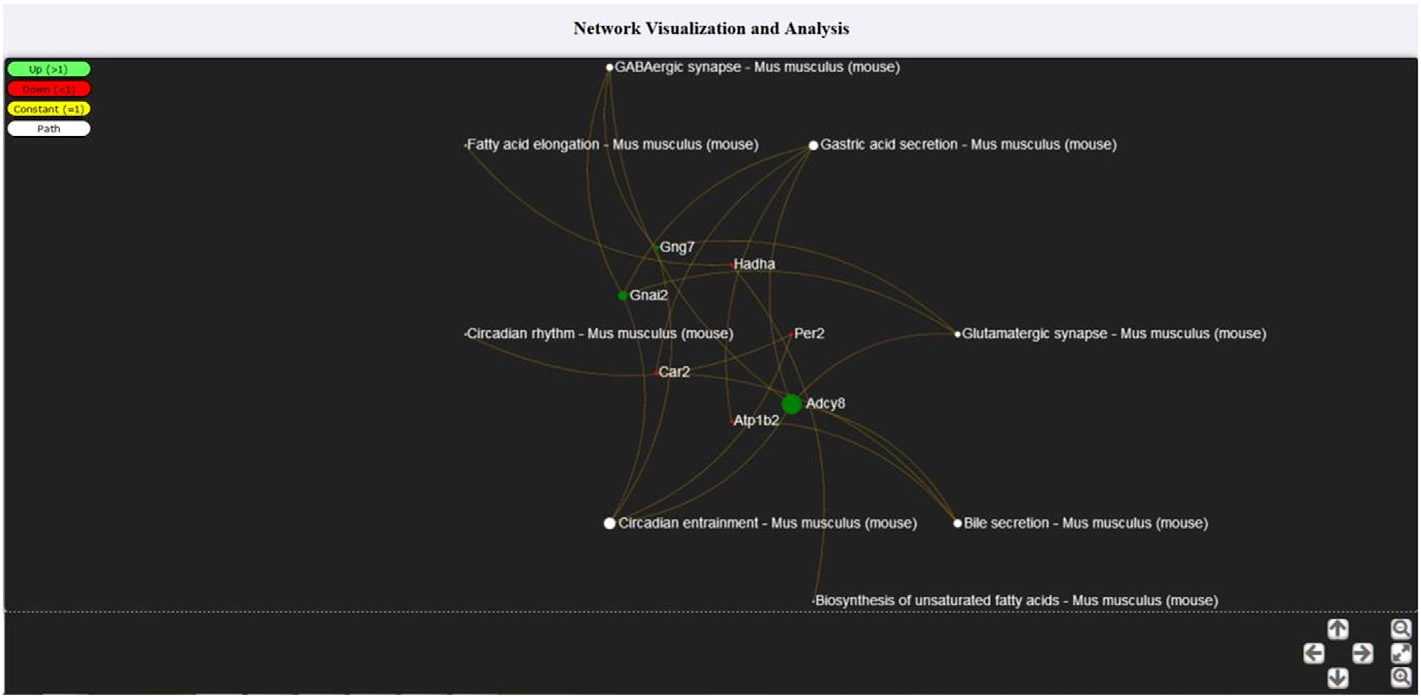
In the present screenshot, a sample gene–pathway interaction network is illustrated. Genes (inner circle) Gng7, Gnai2 and Adcy8 are up-regulated (green color), while Car2, Atp1b2, Per2 and Hadha are down-regulated (red color). By clicking on a pathway (outer circle with white colors), genes that found on the selected pathway are highlighted and vice-versa.

For the case of genes–pathways mapping (see Fig. 3), visualization was implemented in a circular layout with two concentric circles, the inner one for the genes and the outer one for the KEGG pathways. Colors on the specific graph corresponded to up- (red)/down- (green) regulated genes, constant (yellow) and KEGG pathways (white). If a gene is found on a specific KEGG pathway, then an edge between the gene and the pathway is depicted, as shown in Fig. 3.

### 3.3 Case study

*KENeV* is showcased here using as input a well defined list of genes that has been related to Type I Diabetes Mellitus and was found deposited in the *Autworks* database (http://autworks.hms.harvard.edu/) [17]. *Autworks* began as a cross-disease biology web portal in order to store genes lists that have been found associated with autism or related disorders. Currently, it contains ~ 2300 additional disorders that can be compared with autism in terms of common gene networks beneath disorders.

**Fig. 4:**
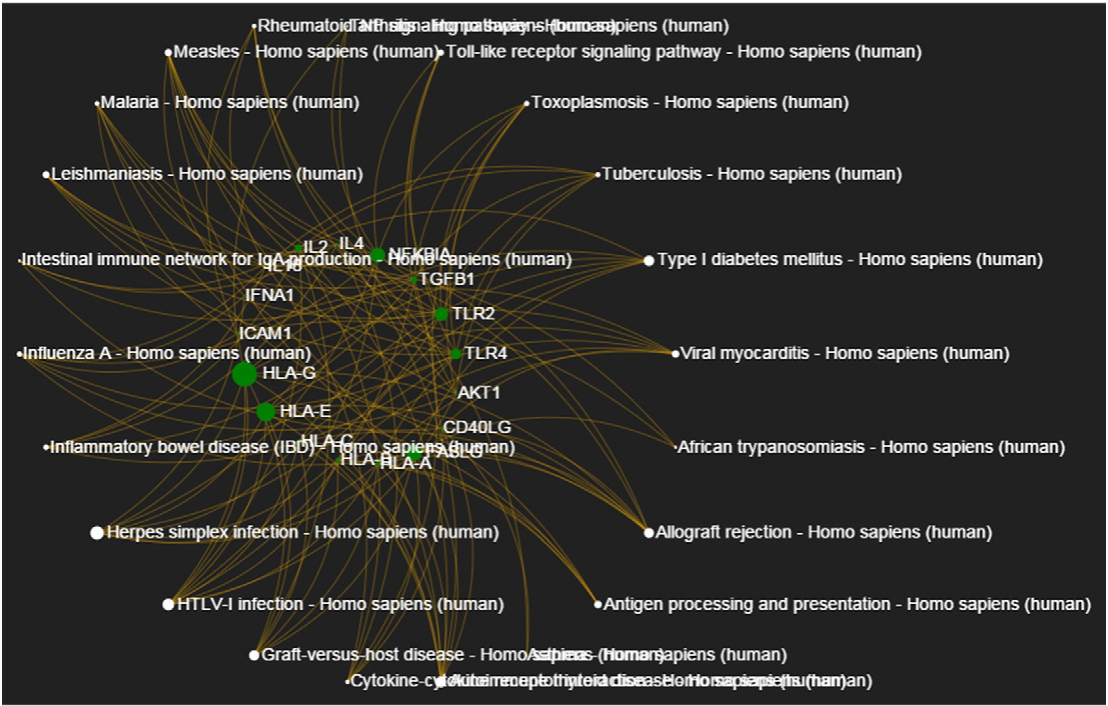
Genes to pathways mapping as constructed by *KENeV* for the analysis of the Type I Diabetes Mellitus gene list.

**Fig. 5:**
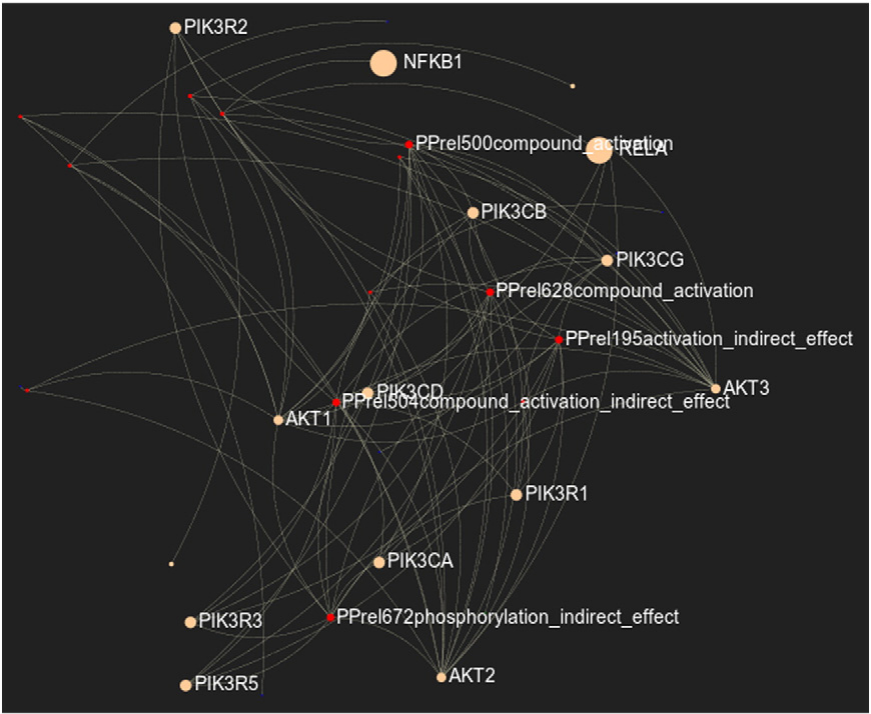
Screenshot of a signaling network instance using the Type I Diabetes Mellitus gene list, showing the cross-talk between the NF-kB/RelA and PI3K pathways.

**Fig. 6:**
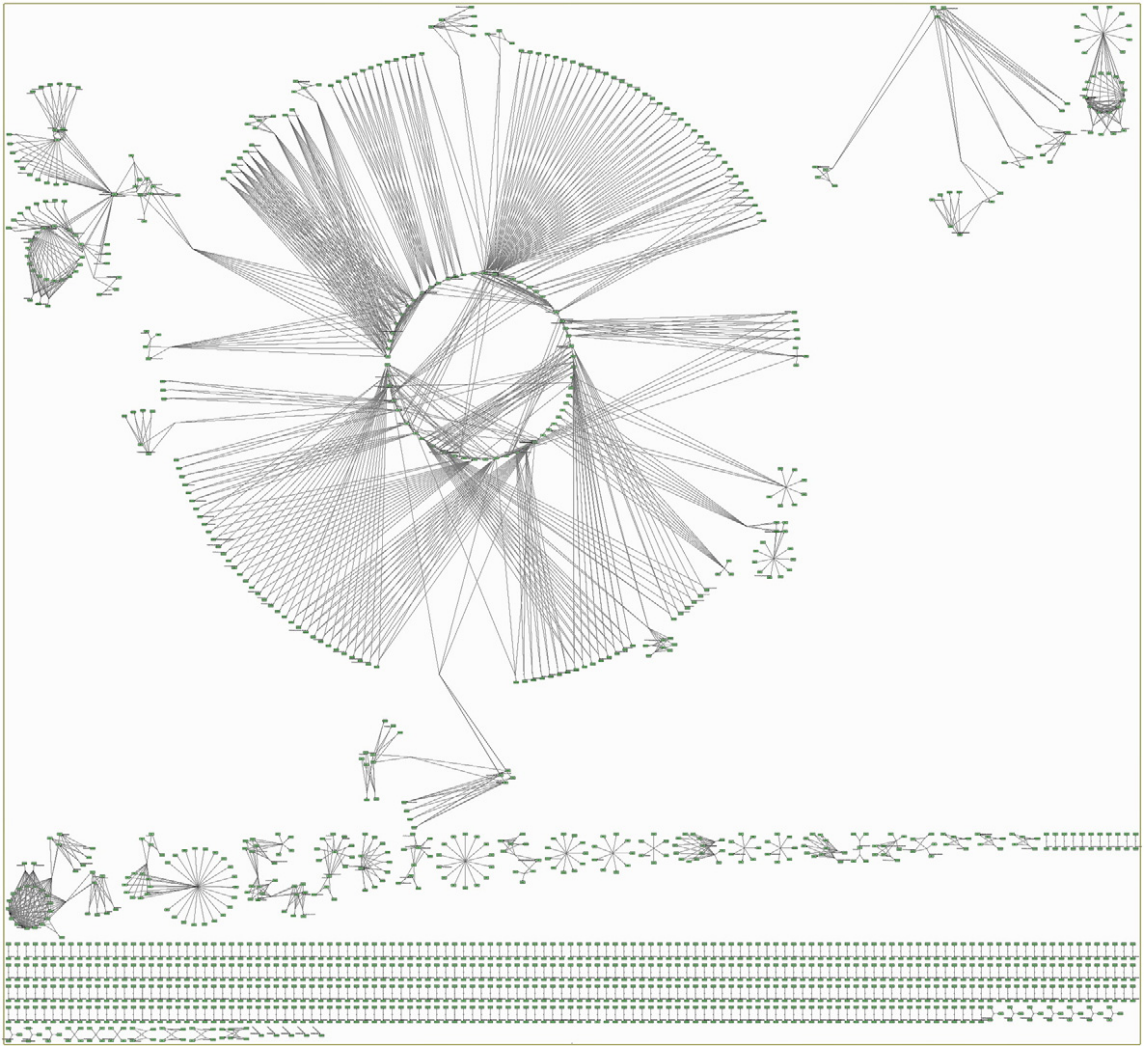
Screenshot of the merged signaling network imported in *CellDesigner* in SBML format.

The list of 407 genes associated to Type I Diabetes Mellitus was submitted to *KENeV* and the workflow was executed up to the visualization module (*p*-value threshold: 0.01, bootstrap distribution cutoff percentage: 90%). Table 1 reports the enriched KEGG pathways extracted by *KENeV* inorder of statistical significance, based on the bootstrap extracted *p*-value (see last column). Some interesting pathways are related to autoimmune disorders and inflammation, while the pathway for the disease itself appears first in the output. Fig. 4 presents the genes to pathways mapping as constructed by the tool. An instance of the signaling network constructed by *KENeV*, focusing on the cross-talk between the NF-kB/RelA and PI3K pathways, is shown in Fig. 5. These pathways are known to control, among various cellular processes, inflammation and metabolism and they are implicated in metabolic and immunity-related diseases [23], [24]. Finally, a screenshot of the merged signaling network imported in SBML format in *CellDesigner* [22] is shown in Fig. 6.

## 4 Discussion

### 4.1 Comparison with similar tools

There are several software tools that allow the retrieval, visualization and/or the analysis of KEGG pathways. However they all have different features and orientations and none combines all key features of *KENeV* in a fully automated integrative online application, namely pathway enrichment analysis, automatic pathway retrieval, handling of metabolic and signaling pathways, handling of KGML inconsistencies, SBML conversion, SBML merging and visualization of the super-pathways, including highlighting of the differentially expressed genes.

For instance, two available KEGG pathway converters, *KEGG2SBML* and *KGML2BioPAX* [25] translate the KGML format to SBML and BioPAX formats respectively. However, they do not perform any handling of the KGML data correction of reactions and entries annotation enhancement, whereas they cannot handle signaling KEGG pathways. Also, they perform simple one-to-one KGML conversions and they cannot be used for complex networks construction.

*Pathview* [26] is an *R*/*Bioconductor* package, which provides KEGG pathway visualization, with very useful features, for instance automatic pathway retrieval, biochemical data modification and enhancement and integration of biological data provided by the user (gene expression, protein expression, metabolite level, genetic association, genomic variation, literature records etc.). However, *Pathview* does not include functions for merging multiple pathways into super-networks.

*KEGGgraph* [27] is another toolbox implemented in *R* and *Bioconductor*, which enables KGML data retrieval, parsing and visualization. Although this package supports merging of multiple graphs (metabolic and signaling), all functionalities are performed manually through specific R commands, and thus the toolbox is not intended to be used automatically by non programming experts.

*KEGGParser* [28] is a KEGG pathway semiautomatic parsing, editing and illustrating tool which is based on *MATLAB* functions, and on Bioinformatics and Image Processing toolboxes. *KEGGParser*'s advantage is the automatic correction of inconsistencies between KGML files and static pathway maps.

A more thoroughly developed KEGG pathway illustrator is *KEGGTranslator* [29], an application for KGML data visualization and conversion to multiple formats. *KEGGTranslator* performs multi layered correction/augmentation of KGMLs biological data, such as the removal of examined-organism unrelated nodes, the completion of reactions, the annotation of each entry with multiple identifiers and the addition of stoichiometric information. Additionally, it provides a functional graphical user interface and translates KGML files into various file formats (SBML, BioPAX). These output files can be used for simulation analysis due to the integrated stoichiometric information.

Another web application is *Pathway Projector* [30], which provides the illustration of metabolic pathways participating in KEGG Atlas, visualized in a Zoomable User Interface (ZUI). Additionally, it offers various functionalities, such as the simultaneous implementation of various “-omics” experimental data. However *Pathway Projector* is not able to visualize signaling and gene regulation networks. Furthermore, *Pathway Projector* does not extract pathway data in an exchangeable format such as SBML and BioPAX.

*KEGG Converter* [31], automatically retrieves, converts to SBML and merges KEGG pathways. However, as it is a simulation-oriented tool, it handles only metabolic pathways, it does not provide any automatic visualization and it is not integrated with any pathway enrichment analysis.

*PathwayLinker* [32] is an online tool that can provide estimates for possible signaling effects of modifying single proteins or selected groups of proteins (e.g., by microRNAs or as drug targets). It integrates protein–protein interaction and signaling pathway data from several sources, thus reducing any manual literature mining, and provides visualization features. It differs from *KENeV* in that it does not perform KEGG pathway enrichment calculations and pathways prioritization, and does not provide visualizations for the metabolic network.

With respect to the pathway over-representation analysis, there are tools that include the KEGG pathways as canonical pathway backgrounds, such as *DAVID* [33], *GeneTrail* [11] and *Webgedstalt* [34]. A direct comparison of *KENeV* to other gene-enrichment tools given the results/lists of KEGG pathways is out of the scope of this manuscript and could not be adequately informative for a thorough comparison. Different tools may provide different or re-arranged lists of pathways due to the different timing in updating the KEGG database (*KENeV* updates on a weekly basis the KEGG database). In terms of functionality, we note here that the following derived from comparing *KENeV* to *DAVID*, *Webgestalt* and *GeneTrail*: *KENEV* does not require a least number of genes per KEGG pathway since bootstrapping excludes such pathways from being reported in the final list of pathways. *KENeV* provides the bootstrapping method instead for providing the user the choice for a series of *p*-value correction methods (user could be unfamiliar to such methods). Importantly, *KENEV* merges pathways into super-pathways and provides all visualization features described. However, we provide in an .xls file (provided as Supplementary data) the lists of significantly enriched KEGG pathways derived when the list of 407 genes associated to Type I Diabetes Mellitus (submitted to *KENeV* as described in the Results section) was uploaded to *DAVID*, *Webgestalt* and *GeneTrail* tools. It can be observed that *KENeV* provides a shorter list of pathways, which is however similar in the firstly prioritized KEGG pathways to the longer ones obtained by the other tools.

Overall, the majority of the aforementioned tools, except from *KEGGgraph*, *KEGG Converter* and *PathwayLinker*, cannot perform multiple pathway integration into a merged network. Another drawback, specifically concerning users without any experience in programming, is that some tools are available as programming packages or simple scripts, instead of automated web applications, in contrast to *KENeV*, which enables the automated reconstruction of both signaling and metabolic enriched networks, using as only input a list of gene identifiers with their respective fold changes.

### 4.2 Future work

In the current version, the application is oriented towards visualization and therefore does not deliver stoichiometric-enabled SBML files for simulation purposes. Nevertheless, the KEGG maps are themselves designed for visualization purposes and hence they are not consistent for simulation. Notably, they contain gaps whereas they do not contain neither the necessary stoichiometry, nor the reactions' cofactors, which are essential for the oxidoreduction and energy balance. This information is however separately available in the *KEGG Reaction* database and it will be used for a future implementation of the application that will derive a simulation-enabled metabolic SMBL file, in addition to the current ones that are enabling visualization. Important steps towards this direction are the effective handling of inconsistencies in matter of pathway gaps, reaction mass balance and missing co-factor information.

Additionally, a merged metabolic/signaling hyper-network can be derived from the integration of metabolic and signaling sub-networks, provided the overcoming of a number of challenges, with respect to the expected high network complexity, especially the handling of complex protein–protein interactions presenting high connectivity. Finally, more interaction databases, for instance *MSigDB* [2] and *Reactome* [35], will be exploited in order to enrich the pathway information.

## 5 Conclusion

Pathway enrichment analysis and visualization approaches provide valuable tools for the interpretation of genomics experimental results, typically characterized by increased dimensionality and complex connectivity. From this perspective, the *KENeV* web application presented here enables the automatic execution of a workflow that combines pathway enrichment analysis, reconstruction of the enriched metabolic and signaling networks, and finally instant interactive visualization of the obtained networks. In conclusion, the application provides an effective tool for the interpretation of the underlying biological mechanisms, which are detected by genomic experiments that derive a subset of genes with differential expression among various conditions.

## Figures and Tables

**Table 1:** Enriched KEGG pathways as extracted by *KENeV* for the analysis of Type I Diabetes Mellitus gene list.

Rank	Term ID	Term description	Hypergeometric *p*-value	Enrichment	Bootstrap *p*-value
1	path:hsa04940	Type I diabetes mellitus — *Homo sapiens* (human)	5.86E-13	28/45	4.10E-05
2	path:hsa05320	Autoimmune thyroid disease — *Homo sapiens* (human)	2.78E-12	23/54	7.75E-05
3	path:hsa05321	Inflammatory bowel disease (IBD) — *Homo sapiens* (human)	3.36E-12	33/67	1.13E-04
4	path:hsa05164	Influenza A — *Homo sapiens* (human)	3.54E-12	40/177	1.47E-04
5	path:hsa04668	TNF signaling pathway — *Homo sapiens* (human)	4.84E-12	27/110	1.82E-04
6	path:hsa05332	Graft-versus-host disease — *Homo sapiens* (human)	5.57E-12	21/43	2.16E-04
7	path:hsa05145	Toxoplasmosis — *Homo sapiens* (human)	5.82E-12	31/120	2.50E-04
8	path:hsa05310	Asthma — *Homo sapiens* (human)	5.85E-12	18/32	2.84E-04
9	path:hsa05162	Measles — *Homo sapiens* (human)	6.27E-12	31/134	3.18E-04
10	path:hsa05152	Tuberculosis — *Homo sapiens* (human)	6.53E-12	40/179	3.53E-04
11	path:hsa05330	Allograft rejection — *Homo sapiens* (human)	7.27E-12	25/39	3.88E-04
12	path:hsa04612	Antigen processing and presentation — *Homo sapiens* (human)	7.33E-12	22/79	4.23E-04
13	path:hsa04672	Intestinal immune network for IgA production — *Homo sapiens* (human)	1.01E-11	21/49	4.57E-04
14	path:hsa05144	Malaria — *Homo sapiens* (human)	1.01E-11	22/49	4.91E-04
15	path:hsa05323	Rheumatoid arthritis — *Homo sapiens* (human)	1.13E-11	27/91	5.25E-04
16	path:hsa05168	Herpes simplex infection — *Homo sapiens* (human)	1.16E-11	46/186	5.59E-04
17	path:hsa05143	African trypanosomiasis — *Homo sapiens* (human)	1.39E-11	16/34	5.92E-04
18	path:hsa05140	Leishmaniasis — *Homo sapiens* (human)	1.87E-11	33/74	6.24E-04
19	path:hsa04060	Cytokine–cytokine receptor interaction — *Homo sapiens* (human)	2.09E-11	48/265	6.60E-04
20	path:hsa04620	Toll-like receptor signaling pathway — *Homo sapiens* (human)	5.27E-11	25/106	6.91E-04
21	path:hsa05166	HTLV-I infection — *Homo sapiens* (human)	7.18E-11	42/261	7.26E-04
22	path:hsa05416	Viral myocarditis — *Homo sapiens* (human)	2.25E-10	18/60	7.58E-04

## Appendix A Supplementary data

Results of KEGG pathway enrichment analysis of the Type I Diabetes Mellitus gene list, using KENeV, DAVID, Webgestalt and GeneTrail.
